# Supplementary material for: miRNA Predictors of Pancreatic Cancer Chemotherapeutic Response: A Systematic Review and Meta-Analysis
Source: Cancers (Basel). 2019 Jun 27;11(7):900. doi: 10.3390/cancers11070900 (PMC6678460; doi:10.3390/cancers11070900)
Supplement: Supplementary file 1 [file cancers-11-00900-s001.pdf]

Supplementary Materials: miRNA Predictors of Pancreatic Cancer Chemotherapeutic Response: A Systematic Review and Meta-Analysis

Madurantakam Royam Madhav, Rithika Ramesh, Ritika Shanker, Shanthi Sabarimurugan, Chellan Kumarasamy, Nachimuthu Ramesh, Kodiveri Muthukalianan Gothandam, Siddharta Baxi, Ajay Gupta, Sunil Krishnan and Rama Jayaraj

Table S1. The search strategy.

**1**	"Pancreatic" OR "Pancreatic cancer" [Title]
**2**	"Pancreatic" OR "Pancreatic cancer" [Title] OR "miRNA" or "microRNA" [Title]
**3**	"Pancreatic" OR "Pancreatic cancer" [Title] OR "Drug resistance" [Title]
**4**	"Pancreatic" OR "Pancreatic cancer" [Title] OR "Chemoresistance" [Title]
**5**	"Pancreatic" OR "Pancreatic cancer" [Title] OR "Chemosensitivity" [Title]
**6**	"Pancreatic" OR "Pancreatic cancer" [Title] OR "miRNA" OR "microRNA" [Title] OR "Drug resistance" [Title]
**7**	"Pancreatic" OR "Pancreatic cancer" [Title] OR "miRNA" OR "microRNA" [Title] OR "Chemoresistance" [Title]
**8**	"Pancreatic" OR "Pancreatic cancer" [Title] OR "miRNA" OR "microRNA" [Title] OR "Chemosensitivity" [Title]
**9**	"Pancreatic" OR "Pancreatic cancer" [Title] OR "miRNA" OR "microRNA" [Title] OR "Chemoresistance" [Title] OR "Chemosensitivity" [Title]

Table S2. Quality assessment of the included studies.

S.No	Criteria	Bad (0–33%)	Satisfactory (33–66 %)	Good (67–100%)
**1**	The objective of this paper stated	-	-	43 Studies
**2**	Study population clearly specified	-	-	43 Studies
**3**	Participation rate of eligible persons at least 50%	-	-	43 Studies
**4**	Eligibility criteria	-	-	43 Studies
**5**	Sample size justification	-	-	43 Studies
**6**	miRNA Exposure assessed before outcome measurement	-	12 Studies	31 Studies
**7**	Timeframe sufficient for the patients (OS, DFS or MFS)	NA	NA	NA
**8**	Different levels of the exposure of interest (mode of treatment)	-	10 Studies	33 Studies
**9**	Exposure measures and assessment (staging of cancer, TNM)	-	3 Studies	40 Studies
**10**	Repeated exposure assessment	-	-	12 Studies
**11**	Outcome measures (HR, and CI)	-	-	13 Studies
**12**	Blinding of outcome assessors	NA	NA	NA
**13**	Follow-up rate	-	41 studies	2 Studies
**14**	Statistical analysis	-	-	43 Studies
	**Total Selected studies**	**0**	**0**	**43**
